# Neuropsychiatric Phenotype and Treatment Challenges in 47,XYY Syndrome: A Narrative Review with a Case Series of Adolescents

**DOI:** 10.3390/brainsci16020232

**Published:** 2026-02-15

**Authors:** Maria Giulia D’Acunto, Chiara Bosetti, Deianira Rinaldi, Marika Ricci, Stefano Berloffa, Gabriele Masi, Maria Mucci

**Affiliations:** 1 Department of Developmental Neuroscience, IRCCS Stella Maris Foundation, Calambrone, 56128 Pisa, Italy; gdacunto@fsm.unipi.it (M.G.D.); chiara.bosetti@fsm.unipi.it (C.B.); deianira.rinaldi@fsm.unipi.it (D.R.); marika.ricci@fsm.unipi.it (M.R.); gabriele.masi@fsm.unipi.it (G.M.); maria.mucci@fsm.unipi.it (M.M.); 2 Department of Clinical and Experimental Medicine, University of Pisa, 56122 Pisa, Italy

**Keywords:** 47,XYY syndrome, Jacobs syndrome, neurodevelopmental disorders, intellectual disability, emotional and behavioral dysregulation, psychiatric comorbidity, pharmacoresistance, antipsychotic treatment, adolescent psychiatry

## Abstract

**Highlights:**

**What are the main findings?**

- 47,XYY syndrome is associated with a broad and heterogeneous neuropsychiatric phenotype.
- Increased risks include language impairment, executive dysfunction, ADHD and autism spectrum traits.

**What are the implications of the main findings?**

- Psychiatric vulnerability appears to rise during adolescence, with higher rates of mood and psychotic disor-ders.
- Case series findings suggest progressive behavioral dysregulation and possible pharmacoresistance in a sub-set of adolescents.
- Long-term, multidisciplinary monitoring is essential to improve clinical outcomes.

**Abstract:**

Background: 47,XYY syndrome is a relatively common sex chromosome aneuploidy that remains largely underdiagnosed. While its somatic phenotype is often mild, growing evidence indicates a substantial burden of neurodevelopmental and psychiatric morbidity. However, the characterization of the neuropsychiatric phenotype across development, particularly during adolescence, and the associated treatment challenges remain incomplete. Objectives: To provide a comprehensive narrative review of the neuropsychiatric phenotype of 47,XYY syndrome and to illustrate clinical complexity and treatment response through a case series of adolescents. Methods: A narrative review of the literature was conducted focusing on genetics, neurodevelopmental and psychiatric features, neuroimaging and neurophysiology findings, clinical course, and management strategies in 47,XYY syndrome. This review is complemented by a case series of adolescents with confirmed 47,XYY karyotype, evaluated for developmental history, psychiatric comorbidity and response to pharmacological and non-pharmacological interventions. Results: The literature consistently describes increased risks of language impairment, executive dysfunction, ADHD, autism spectrum traits, and emotional and behavioral dysregulation in males with 47,XYY syndrome. Psychiatric vulnerability appears to increase during adolescence and adulthood, with elevated rates of mood, psychotic, and substance use disorders. The presented cases illustrate a convergent clinical trajectory marked by early developmental delays, progressive behavioral dysregulation in adolescence and limited or inconsistent response to multiple classes of psychotropic medications, suggesting a pattern of pharmacoresistance in a subset of patients. Conclusions: 47,XYY syndrome is associated with a distinct and heterogeneous neuropsychiatric phenotype that extends beyond early neurodevelopmental disorders. Early diagnosis alone may be insufficient to prevent severe psychiatric outcomes, highlighting the need for long-term monitoring and integrated, multidisciplinary management. Further research is required to identify early predictors of high-risk trajectories and to optimize treatment strategies for this population.

## 1. Introduction

47,XYY syndrome, also known as Jacobs syndrome, is a sex chromosome aneuploidy affecting approximately 1 in 1000 male births. Despite its relatively high prevalence, the condition remains markedly underdiagnosed, with most affected individuals unaware of their karyotype throughout life. This diagnostic gap is largely attributable to the absence of distinctive physical features and the wide variability of clinical presentation.

Historically, clinical attention to 47,XYY syndrome focused on early and often stigmatizing assumptions regarding aggression and criminal behavior, which have since been robustly refuted. Contemporary research has shifted the focus toward a more nuanced understanding of the syndrome, emphasizing neurodevelopmental vulnerability, cognitive and language impairments, and increased risk for psychiatric disorders. Advances in genetics, neuroimaging, and population-based registry studies have further contributed to redefining 47,XYY syndrome as a condition primarily characterized by neuropsychiatric complexity rather than overt somatic pathology.

Accumulating evidence indicates that males with 47,XYY syndrome are at increased risk for language disorders, executive dysfunction, attention-deficit/hyperactivity disorder (ADHD), and autism spectrum disorder (ASD). Beyond childhood, large-scale epidemiological studies have documented elevated rates of mood disorders, schizophrenia spectrum disorders, substance use disorders, and increased overall morbidity and mortality. Notably, adolescence appears to represent a critical developmental window during which psychiatric symptoms may intensify, yet this phase remains relatively underrepresented in the literature.

Although early genetic diagnosis has become more common with the widespread use of non-invasive prenatal screening, clear syndrome-specific management guidelines are lacking. Pharmacological treatment is typically guided by standard approaches to neurodevelopmental and psychiatric disorders, but clinical experience suggests that treatment response may be variable and, in some cases, limited. The extent to which pharmacoresistance or complex treatment needs characterize a subset of individuals with 47,XYY syndrome has not been systematically explored.

The aims of the present article are therefore twofold: first, to provide a comprehensive narrative review of the current literature on the neuropsychiatric phenotype, clinical course, and management of 47,XYY syndrome; and second, to complement this review with a case series of adolescents, highlighting real-world clinical trajectories and treatment challenges. By integrating population-level evidence with detailed clinical observations, this work seeks to contribute to a more refined understanding of the neuropsychiatric manifestations of 47,XYY syndrome and to inform future clinical and research directions.

### 1.1. Definition, Epidemiology, and Genetics of the Syndrome

47,XYY syndrome is a sex chromosome aneuploidy (SCA) occurring in approximately 1 in 1000 male births, making it one of the most common chromosomal duplications [1,2]. Despite its relatively high incidence, the condition remains markedly underdiagnosed; clinical identification rates are estimated at only 15–18%, largely due to the subtlety of the physical phenotype [3,4]. Jodarski et al. [4] highlighted this diagnostic gap by referencing UK Biobank data from Zhao et al. [5], which showed that 99% of identified 47,XYY men were unaware of their genetic status.

Genetic studies indicate that the syndrome most commonly arises from a spontaneous nondisjunction error during paternal meiosis II, or less frequently from postzygotic mitotic errors leading to mosaicism, such as 46,XY/47,XYY [2,3]. Tallaksen et al. [6] proposed that the phenotype is largely driven by overdosage of genes located in the pseudoautosomal region 1 (PAR1), including *SHOX*, which is directly implicated in the characteristic tall stature.

Moreover, Larsen et al. [7] recently applied novel network-based approaches to demonstrate that Y-chromosome dosage effects can be clearly distinguished from X-dosage effects in males, supporting the existence of a distinct genetic architecture underlying psychopathology in 47,XYY syndrome. The regulatory role of specific genes, such as *ZFX*, which organizes co-expression networks involving autosomal genes, has also been proposed as a potential modifier of the clinical phenotype [6].

With respect to genomic variability, Mountford et al. [8] examined the burden of additional copy number variations (CNVs) across the genome and concluded that, although common, this burden does not reliably predict the severity of the neurodevelopmental phenotype in SCT children. These findings challenge the “two-hit” or “double-hit” hypothesis, which posits that secondary genetic variants exacerbate the effects of the primary trisomy. Finally, Samango-Sprouse et al. [9] reported a marked shift in the timing of diagnosis, noting that the widespread implementation of non-invasive prenatal screening (NIPS) has substantially increased rates of prenatal detection.

### 1.2. Somatic Phenotypic Characteristics of 47,XYY Syndrome

The somatic phenotype of 47,XYY syndrome is generally mild and highly variable, lacking a pathognomonic pattern, in contrast to other sex chromosome trisomies [2,3,10].

Tall stature represents the most consistently reported somatic characteristic [11,12]. Birth weight and length are usually within the normal range, whereas accelerated growth—typically emerging after six years of age—results in above-average height during adolescence and adulthood in a substantial proportion of individuals [11,13]. Despite increased stature, body proportions are generally preserved, and disproportionate growth or skeletal dysplasia are not defining features of the syndrome.

In a cohort of 92 boys with 47,XYY syndrome (mean age 9.6 years), Bardsley et al. [11] reported body weight within the normal range overall, although a tendency toward central adiposity was observed. In the same cohort, macrocephaly was reported in 33% of individuals, hypertelorism in 59%, fifth-finger clinodactyly in 52%, hypotonia in 63%, pes planus in 52%, and intention tremor in 43%. Despite their relatively high prevalence in clinically ascertained samples, these features lack sufficient consistency and specificity to serve as reliable diagnostic markers [11,12]. Additional musculoskeletal anomalies, such as genu valgum, have been described. An increased prevalence of ophthalmological disorders, including myopia, astigmatism, and glaucoma, has also been reported. From a neurological perspective, an elevated risk of epilepsy, febrile seizures, and sleep disorders, including obstructive sleep apnea, has been observed [3].

Pubertal onset and progression in males with 47,XYY syndrome are generally within the normal male range; however, increased testicular volume relative to age is frequently observed during adolescence, with macroorchidism reported in a substantial subset of individuals [11]. Circulating testosterone concentrations are typically appropriate for age. Although testicular function is often considered largely normal, Davis et al. [14] identified subtle impairments beginning in childhood, characterized by significantly reduced inhibin B levels and elevated anti-Müllerian hormone concentrations compared with controls. These findings suggest a potential risk for subfertility in adulthood, supporting the need for early semen analysis and reproductive counseling during adolescence [4,14].

### 1.3. Habitual Course, Mortality, and Recommended Medical Follow-Up

The clinical course of 47,XYY syndrome is characterized by considerable phenotypic variability. Systemic morbidity is significantly increased; nationwide registry studies conducted by Berglund et al. [3] demonstrated more than a twofold elevation in the risk of hospital diagnoses across 14 of 18 ICD-10 chapters, including disorders of the respiratory and circulatory systems.

Davis et al. [15] performed a large-scale meta-analysis involving 1.5 million participants from the Million Veteran Program and the UK Biobank, revealing that 47,XYY syndrome is associated with a markedly increased risk of vascular conditions, such as chronic venous insufficiency (OR 5.6) and deep vein thrombosis (OR 5.5). Mortality is also substantially elevated, with life expectancy estimated to be approximately 10 years shorter than that of XY peers, primarily due to respiratory disease, circulatory disorders, and congenital malformations [1,2].

Metabolic monitoring is essential, as individuals with 47,XYY syndrome frequently exhibit an unfavorable metabolic profile, including increased risks of obesity and type 2 diabetes [12,15].

Routine medical follow-up should also address dental abnormalities, such as macrodontia and an increased risk of caries, which may be related to triplicate expression of the *AMELY* gene or reduced salivary flow secondary to psychotropic medication use [3]. Rare but clinically relevant ophthalmological findings have been described. Rubalcava-Soberanis et al. [16] reported a 4-year-old child with microspherophakia and phacomorphic glaucoma, while other cases have documented optic nerve enlargement or retinal atrophic holes. Overall, comprehensive and longitudinal follow-up is crucial, as earlier diagnosis appears to be associated with a lower cumulative comorbidity burden [12].

### 1.4. Neuropsychological Phenotype, Neuroimaging, Neurophysiology, and Neurodevelopmental Comorbidities

The neurodevelopmental phenotype of 47,XYY syndrome is characterized by an increased risk of language impairment, cognitive difficulties, and behavioral challenges [10,17]. Overall intellectual functioning typically falls within the normal to low-average range; however, verbal IQ is consistently more affected than performance IQ [11,18]. Neurodevelopmental comorbidities are highly prevalent, with ADHD symptoms reported in up to 76% of 47,XYY males and autism spectrum disorder diagnosed in approximately 19–40% of cases [18,19,20]. Schaffer et al. [21] demonstrated that 47,XYY syndrome is associated with greater penetrance of social and attentional difficulties compared with Klinefelter syndrome.

Early deficits in social cognition, including impairments in theory of mind and social orienting, can be observed from toddlerhood and tend to intensify over time, a phenomenon described as “growing into deficit” [22]. Using eye-tracking paradigms, Urbanus et al. [23] showed that children with 47,XYY syndrome spend significantly less time fixating on the faces and eyes of social interaction partners. In parallel, Roybal et al. [24] identified a distinctive age-related increase in the severity of social difficulties specific to the 47,XYY karyotype.

Neuroimaging studies have reported increased total gray and white matter volumes [25], alongside regional reductions in frontotemporal areas implicated in language processing [26]. From a neurophysiological perspective, Bloy et al. [27] described significant delays in auditory evoked responses (M50) in the left hemisphere, while Matsuzaki et al. [28] identified abnormal auditory mismatch fields that correlate with the severity of language impairment. Roberts et al. [29] further reported reduced levels of the inhibitory neurotransmitter GABA in the temporal lobe, together with an absence of the typical association between GABA and testosterone levels observed in XY controls.

Epilepsy represents a secondary clinical feature within the 47,XYY syndrome phenotype. In the largest available cohort study, seizures were reported more frequently in individuals with 47,XYY syndrome than in the general pediatric population, although precise prevalence rates were not provided [11]. Case reports and small series further describe focal epilepsy phenotypes, often resembling benign epilepsy with centrotemporal spikes, with occasional atypical evolution such as continuous spikes and waves during slow sleep [30,31]. Case reports have described a range of atypical presentations, including Asperger’s disorder in a high-functioning adolescent [32], deficits in spatial memory encoding [33], and early-onset bipolar disorder with psychotic features [34]. Across studies, early diagnosis consistently emerges as the strongest predictor of improved outcomes through timely and targeted intervention [9,11].

### 1.5. Psychiatric Comorbidity Beyond Neurodevelopmental Disorders and Its Impact on Clinical Course

Beyond neurodevelopmental disorders, 47,XYY syndrome is associated with a clinically significant burden of psychiatric comorbidity, with psychiatric vulnerability becoming more apparent during adolescence and adulthood [3]. Mood disorders, particularly anxiety and depression, in individuals with 47,XYY syndrome are more frequently reported during adolescence and adulthood than in childhood [21]. While population-based registry studies indicate an increased risk of major depressive disorder in adulthood, pediatric clinical cohorts do not consistently show elevated depressive symptoms on standardized measures compared with normative samples [3,11,35]. This pattern suggests that depressive symptomatology may not represent a core feature of the childhood phenotype but may emerge later in development [11].

From a developmental perspective, converging population-based and clinical data indicate that psychiatric morbidity in 47,XYY syndrome becomes more apparent during adolescence and early adulthood, with higher rates of mood and psychotic disorders reported at later developmental stages [3,21]. Neurocognitive and adaptive difficulties commonly described in childhood and adolescence may become more clinically salient as functional demands increase with age [36]. In addition, Urbanus et al. [10] emphasized that limited recognition of early neurobehavioral vulnerabilities may delay appropriate support, potentially contributing to increased psychiatric burden later in development.

In addition to affective disorders, large nationwide registry studies have documented significantly elevated rates of severe psychiatric conditions, including schizophrenia spectrum disorders and bipolar disorder, as well as a higher prevalence of adjustment- and stress-related disorders and substance use disorders, particularly alcohol-related conditions [3]. Consistent with these population-level findings, single-case reports have documented early-onset bipolar disorder with psychotic features in adolescents with 47,XYY syndrome, illustrating the potential for severe affective and psychotic presentations in this population [34].

Emotional dysregulation, affective lability, and aggressive outbursts have also been described in 47,XYY syndrome; however, behavioral dysregulation is primarily attributed to cognitive and executive deficits rather than intrinsic aggression [37,38]. Early misconceptions linking 47,XYY syndrome to criminality have been refuted, and any increased risk of antisocial behavior appears to be mediated by learning difficulties and challenges in social adaptation [39,40]. More recent analyses have reinforced this conclusion, highlighting the central role of contextual and developmental factors in shaping behavioral outcomes in individuals with 47,XYY syndrome [19,21].

### 1.6. Integrated Management of 47,XYY Syndrome: Psychopharmacological, Rehabilitative, and Caregiver-Focused Interventions

The management of 47,XYY syndrome requires an integrated, multidisciplinary approach combining psychopharmacological treatment, targeted rehabilitative interventions, and structured support for families and caregivers [2,41,42]. To date, no syndrome-specific treatment guidelines have been established; consequently, therapeutic strategies are largely based on standard clinical practice for neurodevelopmental and psychiatric conditions and are tailored to individual developmental profiles and functional needs [36,42]. Early identification facilitates timely intervention and family support, and awareness of early neurobehavioral vulnerabilities may guide preventive rehabilitative and caregiver-focused strategies [41,43].

Pharmacological management in 47,XYY syndrome primarily targets comorbid neuropsychiatric conditions [3]. In a large national clinical cohort, Hall et al. [41] documented higher rates of psychotropic medication use in individuals with 47,XYY syndrome compared with matched controls, including stimulant medications for attention-deficit/hyperactivity disorder, as well as antipsychotic agents and selective serotonin reuptake inhibitors in the context of severe behavioral dysregulation, mood instability, psychotic symptoms, or internalizing disorders.

Evidence from earlier clinical studies supports the use of stimulant medications for ADHD in this population. Tartaglia et al. [20] reported a positive clinical response to stimulant treatment in the majority of children and adolescents with sex chromosome aneuploidies and comorbid ADHD, with a 79% response rate among individuals with XYY syndrome and a relatively low incidence of significant adverse effects. Increased irritability was the most common side effect leading to treatment discontinuation, supporting the use of low starting doses and gradual titration. Early case reports by Ruud et al. [44] similarly described clinical improvement and acceptable tolerability in two boys aged 11 and 12 years with 47,XYY syndrome treated with stimulant medication. Data on non-stimulant medications remain limited, although available observations suggest acceptable tolerability and a potential role when stimulant treatment is contraindicated or poorly tolerated.

Emotional dysregulation or aggression is often managed with atypical antipsychotics, such as risperidone or aripiprazole [3]. Selective serotonin reuptake inhibitors (e.g., fluoxetine, sertraline) are commonly prescribed to address anxiety and depressive symptoms, although controlled trials in 47,XYY syndrome are lacking. Case-based evidence further illustrates the potential complexity of psychopharmacological management. Notably, Randolph et al. [45] reported a case of neuroleptic malignant syndrome during treatment with quetiapine, underscoring the need for careful monitoring and individualized risk–benefit assessment when antipsychotic agents are prescribed.

Non-pharmacological interventions represent a cornerstone of supportive care in 47,XYY syndrome. Speech and language therapy is particularly relevant given the high prevalence of language and communication difficulties, while occupational and physical therapy are frequently indicated to address hypotonia, motor delays, and adaptive functioning [10,41,42]. Educational support is commonly required due to learning difficulties, and behavioral and social skills interventions are essential in light of the elevated prevalence of autism spectrum traits and social-communication difficulties [41].

Structured support for caregivers constitutes a fundamental component of effective treatment planning. Multidisciplinary counseling following prenatal or early postnatal diagnosis can reduce parental distress and facilitate informed decision-making [36,42]. Psychoeducational interventions enhance understanding of the child’s developmental profile and promote adherence to therapeutic and educational strategies, while parent-mediated interventions and caregiver coaching programs may improve coping strategies and reduce caregiver stress, indirectly supporting treatment effectiveness and overall family functioning [2,7].

## 2. Clinical Cases

### 2.1. Materials and Methods

This study employed a retrospective, descriptive case series design based on the longitudinal clinical evaluation of three male adolescents diagnosed with 47,XYY syndrome who were followed at the Psychiatric Emergency Unit of IRCCS Stella Maris Foundation, Scientific Institute of Child Neurology and Psychiatry, Calambrone, 56128 Pisa, Italy

Clinical data were obtained through systematic review of medical charts, including developmental history, neurological and psychiatric assessments, neuropsychological testing, neuroimaging and neurophysiological findings, and detailed pharmacological records. Cognitive functioning was evaluated using standardized age-appropriate instruments (WISC-IV Wechsler Intelligence Scale for Children—Fourth Edition; Pearson, San Antonio, TX, USA or WPPSI-III Wechsler Preschool and Primary Scale of Intelligence—Third Edition; Pearson, San Antonio, TX, USA, while adaptive skills were assessed with the Vineland Adaptive Behavior Scales (VABS-II Vineland Adaptive Behavior Scales—Second Edition; Pearson, San Antonio, TX, USA) when available. Brain MRI (3TMR system, MR750 scanner, GE Healthcare, Chicago IL USA) and EEG (Micromed, Mogliano Veneto Italy 10-20 international system) were performed according to clinical indications and interpreted by board-certified neuroradiologists and neurophysiologists. Genetic testing included standard karyotyping and array-comparative genomic hybridization (array-CGH; Cytogenomics 5.0.2.5/Default analysis method CGH V2 (Agilent Technologies, Santa Clara, CA, USA). Psychiatric diagnoses were based on DSM-5 criteria, supported by multidisciplinary evaluations and behavioral observations across multiple settings. Pharmacological histories were reconstructed through prescription records, hospitalization reports, and caregiver interviews, with treatment response categorized as effective, partially effective, ineffective, or discontinued due to adverse effects. All procedures adhered to the Declaration of Helsinki and were approved by the Ethics Committee of Meyer Children’s Hospital (PA_10/2025). Written informed consent for participation and publication was obtained from all legal guardians.

This case series utilizes illustrative exemplars to bridge the gap between population-level data and real-world clinical practice, specifically highlighting the “growing into deficit” phenomenon. These cases were selected to demonstrate phenotypic convergence across developmental trajectories, the severity spectrum of adolescent psychiatric morbidity, and the distinct challenge of multi-class pharmacoresistance. By providing granular longitudinal evidence, this series complements the narrative review, offering specific insights into the complex treatment needs and refractory outcomes frequently encountered in specialized psychiatric emergency settings.

Pharmacoresistance has been explicitly operationalized in the manuscript, with detailed criteria reported in the following paragraph, reflecting failure across multiple medication classes at adequate dose and duration.

In accordance with the Treatment Recommendations for the Use of Antipsychotics for Aggressive Youth (TRAAY), atypical antipsychotics were considered first-line pharmacological agents for the management of severe aggression in adolescence, with adjunctive mood stabilisers employed in cases of partial response [46,47]. Within this framework, the three adolescents with 47,XYY syndrome and severe destructive and aggressive behaviours were classified as pharmacoresistant based on criteria adapted from the STRATA-G consortium definition of antipsychotic treatment resistance [48]. Specifically, pharmacoresistance was defined by the presence of at least one of the following conditions: (i) exposure to clozapine; (ii) sequential treatment with two different antipsychotics at therapeutic doses—defined as at least the mid-range of the licensed dosage—for a minimum duration of six weeks each; or (iii) persistence of clinically significant aggressive symptoms and associated moderate functional impairment despite adequate antipsychotic treatment. To enhance methodological rigour, all patients were required to have received at least one atypical antipsychotic, at least one typical antipsychotic, and at least one mood stabiliser over the course of treatment, thereby ensuring that treatment resistance was defined only after exposure to pharmacological strategies consistent with established clinical recommendations.

In the present case series, the classification of pharmacological inefficacy or partial benefit reported in Table 1, Table 2 and Table 3 was not based on standardized rating scales. Instead, treatment response was determined through structured clinical judgment by at least two senior child and adolescent neuropsychiatrists with extensive expertise in developmental psychopathology, working within a tertiary-care referral center. These clinical evaluations were informed by longitudinal observation and were systematically integrated with collateral anamnestic information obtained from multiple sources, including primary caregivers, community-based child neuropsychiatrists, and educational professionals.

### 2.2. Common Neurodevelopmental and Behavioral Features Across the Three Cases

Across the three cases, the patients were male adolescents with a genetically confirmed 47,XYY karyotype, referred to a tertiary-care neuropsychiatric center for complex neurodevelopmental and psychiatric presentations. These cases provide a unique opportunity to examine convergent phenotypic patterns in 47,XYY syndrome, highlighting the interplay between early neurodevelopmental vulnerabilities, cognitive challenges, and persistent behavioral dysregulation. All patients showed early developmental vulnerability, with feeding difficulties during infancy and abnormal growth trajectories evolving toward overweight or obesity in later childhood or adolescence. Language development was consistently delayed, requiring early speech therapy, while cognitive functioning ranged from borderline intellectual functioning to moderate intellectual disability.

From early childhood, all patients exhibited significant emotional–behavioral dysregulation, characterized by impulsivity, low frustration tolerance, oppositional behaviors, and prominent anxiety symptoms, often including separation anxiety. Behavioral manifestations were persistent over time and showed limited response to rehabilitative interventions alone, necessitating multiple pharmacological trials. Adaptive functioning was globally impaired in all cases.

### 2.3. Case Report 1

In addition to the shared clinical features described above, Case 1 was characterized by early perinatal complications, including oligohydramnios, neonatal intensive care admission with non-invasive ventilation for respiratory distress, and mild periventricular hyperechogenicity on early neuroimaging. Neurodevelopmental follow-up documented moderate intellectual disability, confirmed by WISC-IV testing.

From late childhood, the clinical course was marked by worsening emotional dysregulation, oppositional behaviors, episodic irritability, and recurrent self- and hetero-aggressive outbursts. Notably, impulsive and inappropriate sexual behaviors emerged, representing a major source of functional impairment.

Endocrinological evaluation revealed reduced gonadotropin and testosterone levels. Between ages 3 and 9, three seizure episodes were reported; EEG recordings showed intermittent sharp waves and fast anterior activity. Brain MRI (2014) revealed a small right frontal subcortical hyperintensity, interpreted as enlarged perivascular spaces, and mild left ventricular predominance, with stable findings on follow-up imaging (2023). Array-CGH confirmed the 47,XYY karyotype and identified a maternally inherited 17p11.2 duplication involving SPECC1, considered of uncertain clinical significance. The pharmacological timeline is reported in Table 1.

### 2.4. Case Report 2

Beyond the common neurodevelopmental profile observed across the case series, Case 2 was notable for a progressive worsening of emotional–behavioral symptoms and the emergence of mood disturbances over time. Although early cognitive functioning was within the mild impairment range, longitudinal assessment revealed a slight decline on repeated WISC-IV testing during adolescence.

The psychiatric phenotype evolved toward severe emotional dysregulation, with irritability, destructive behaviors, paranoid ideation, and ruminative thought patterns. At the most recent hospitalization, the patient met diagnostic criteria for a mood disorder with depressed–dysphoric mood, impulse control disorder, oppositional defiant disorder, and mixed anxiety disorder, within a broader framework of ADHD (combined type) and impaired adaptive functioning.

EEG recordings initially showed anterior fast activity and left frontotemporal sharp waves, later evolving into bilateral centrotemporal epileptiform abnormalities, while brain MRI remained normal. Rehabilitative interventions provided limited benefit, particularly with respect to behavioral control. The pharmacological history is summarized in Table 2.

### 2.5. Case Report 3

Case 3 differed from the other patients by an earlier and more global neurodevelopmental impairment, followed by a comparatively more favorable adaptive trajectory after intensive intervention. Developmental milestones were significantly delayed, including late acquisition of walking, language, and sphincter control. Early evaluations led to a diagnosis of complex developmental disorder with intellectual disability and severe emotional–behavioral dysregulation.

Cognitive testing confirmed mild intellectual disability with pronounced visuographic deficits. The clinical picture was further complicated by severe communication impairments, including phonological and morphosyntactic deficits, impaired pragmatic skills, and occasional neologisms. Behavioral manifestations included hyperactivity-impulsivity, self- and hetero-aggressive behaviors, anxiety disorders, oppositionality, obsessive–compulsive traits, and self-harm.

EEG showed parieto-occipital paroxysmal abnormalities, more evident during sleep. Brain MRI revealed nonspecific T2 white-matter hyperintensities and mild ventricular asymmetry. Following the implementation of intensive, structured educational, rehabilitative, and social interventions, the patient showed improved adaptive functioning and clinical stabilization. A detailed overview of pharmacological interventions is presented in Table 3.

At age 10, educational, rehabilitative, and social interventions were intensified, including individualized support at school and at home, as well as placement in a day centre, resulting in positive outcomes. Since 13.9 years, increased fatigability, oppositionality, and tic-like head movements have been reported. He completed secondary school with special-education support, showing adequate adaptation with occasional behavioral dysregulation. Social and recreational integration remained positive through participation in structured activities and family involvement. Clinical stability was maintained, with preserved routines and no major crises. He is now transitioning to agricultural high school with individualized support, maintaining motivation in inclusive, protected settings.

## 3. Discussion

47,XYY syndrome is a sex chromosome aneuploidy affecting approximately 1 in 1000 live male births, characterized by the presence of an additional Y chromosome and a wide spectrum of neurodevelopmental and psychiatric manifestations [3,11,36]. Although many affected individuals remain undiagnosed due to the absence of distinctive physical phenotypes, the cumulative burden of neurobehavioral comorbidities—particularly language delays, executive dysfunction, and high rates of ADHD, ASD, and mood dysregulation—has become increasingly recognized [17,49].

**Figure 1:**
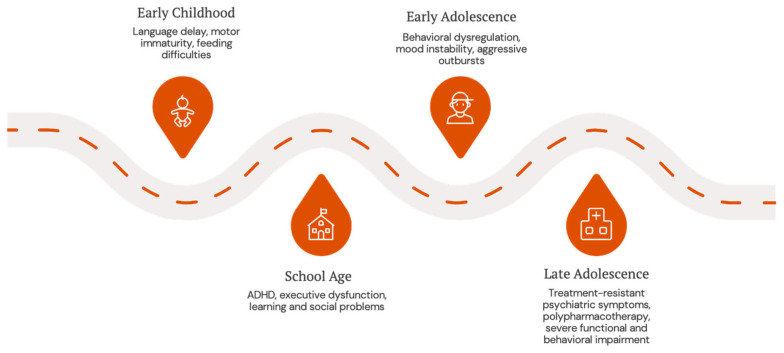
The “growing into deficit” trajectory from early childhood to adolescence.

The three male patients described in this report were all diagnosed with 47,XYY in early childhood and exhibited strikingly similar neurodevelopmental and behavioral trajectories (see Figure 1). All three demonstrated early language delays and motor immaturity, followed by emotional dysregulation, social withdrawal, irritability, and attentional difficulties. Importantly, each patient progressed to complex psychiatric profiles during adolescence, marked by multi-drug resistant behavioral symptoms necessitating polypharmacy regimens and multidisciplinary interventions. This convergence supports the existence of a potential 47,XYY-specific neuropsychiatric phenotype, characterized by early developmental vulnerability, moderate intellectual disability, and escalating psychiatric comorbidity with poor response to standard treatments.

This timeline illustrates the characteristic “growing into deficit” pattern observed across all three clinical cases. Early neurodevelopmental vulnerabilities progressively evolve into complex psychiatric presentations during adolescence, with increasing treatment resistance and functional impairment. Each stage represents a critical window where symptoms intensify and new comorbidities emerge, highlighting the need for continuous monitoring and adaptive intervention strategies throughout development.

Although tall stature, macrocephaly, and hypotonia are frequently reported in 47,XYY individuals [11], these somatic features are rarely diagnostic and are often overlooked. All three patients presented with heights above the 97th percentile, consistent with previous studies implicating SHOX gene overexpression in increased linear growth [21]. Regarding hormonal profile, while circulating testosterone concentrations in 47,XYY syndrome were historically considered appropriate for age, Case 1 represents a clinically significant subgroup rather than a distinct outlier. Although 93% of children in specific pediatric cohorts demonstrate normal ranges, recent meta-analytic data indicate that testosterone levels in 47,XYY individuals are typically lower than those of healthy 46,XY males [11,50]. Case 1’s hormonal profile mirrors recent evidence showing that approximately 38.9% of subjects with 47 XYY syndrome exhibit total testosterone levels below the normal threshold [50].

Nevertheless, it is the neurodevelopmental profile rather than physical traits that provides the most reliable diagnostic clues. Delayed expressive language, low-average to borderline IQ with verbal-performance discrepancies, and early deficits in social reciprocity were prominent in these cases. These observations are consistent with findings from the eXtraordinarY Kids and NIH cohorts, which report high rates of expressive language disorders, verbal apraxia, and pragmatic language delays in 47,XYY individuals [36,49,51].

Beyond language, executive dysfunction—particularly impaired inhibitory control, attentional instability, and working memory deficits—emerged as a key driver of psychiatric vulnerability. All three patients exhibited severe ADHD-like symptoms, including impulsivity, poor frustration tolerance, and attentional lability. These deficits likely contributed to the subsequent emergence of complex behavioral dysregulation, reinforcing the central role of executive function impairment in shaping neuropsychiatric outcomes, such as outcomes related to cognitive, emotional, and behavioral functioning in 47,XYY.

The most clinically relevant feature across all three patients was the presence of multi-class psychiatric pharmacoresistance. Despite extended and structured pharmacological management—including stimulants (methylphenidate), mood stabilizers (valproic acid, lithium carbonate), and multiple antipsychotics (risperidone, aripiprazole, chlorpromazine, olanzapine, clozapine, lurasidone, haloperidol)—therapeutic outcomes were largely unsatisfactory. Medications were often discontinued due to ineffectiveness, paradoxical responses (e.g., increased agitation with methylphenidate), or adverse events (e.g., hyperphagia with risperidone, autonomic symptoms with clozapine). Even combinations of agents, carefully titrated under psychiatric supervision, provided only partial or transient benefit, while emotional and behavioral dysregulation persisted at high intensity, frequently necessitating hospitalization or therapeutic reassessment.

This pattern of broad-spectrum pharmacoresistance may represent a potential clinically significant phenomenon in 47,XYY syndrome that is seldom documented in the literature. Although pharmacotherapy is often reported in cohort studies [36,49], systematic descriptions of non-response are limited. Our cases provide detailed evidence of persistent pharmacologic inefficacy across multiple therapeutic classes, highlighting an important aspect of clinical management that warrants further investigation.

Mechanistically, pharmacoresistance may reflect underlying neurobiological differences inherent to 47XYY syndrome. Structural and functional neuroimaging studies demonstrate increased total brain volume alongside region-specific cortical thinning in frontotemporal and motor regions, with altered frontostriatal connectivity implicated in emotional regulation and behavioral control [17,25]. Integrating recent iPSC-based molecular findings provides a robust neurobiological framework for the “growing into deficit” phenomenon and the complex psychiatric trajectories documented in our case series. Our clinical findings, which track an evolutionary transition from early language delays to severe, multi-class pharmacoresistance during adolescence, are mirrored at the molecular level by the transcriptional dysregulation of pathways essential for neuron projection, axon guidance, and synapse organization [52]. Specifically, the consistent overdosage of the Y-linked gene NLGN4Y—which is significantly upregulated in 47,XYY models and associated with disrupted synaptogenesis and social impairments—aligns with the heightened risk for social deficits and Autism Spectrum Disorder traits exhibited by our three patients, potentially influencing both behavioral phenotype and pharmacodynamic response [17,49,52].

Furthermore, the discovery of a shared transcriptomic signature between Jacobs and Klinefelter syndromes, particularly involving dysregulated voltage-gated ion channel activities, offers a potential biological explanation for the refractory response to standard psychotropic medications observed in our clinical cohort [52]. The identification of a unidirectional UTY-KDM6A regulatory mechanism further underscores a genetically encoded neurobiological instability that may drive the escalating psychiatric morbidity and mood disorders observed during the second decade of life [52]. These omics-based insights reinforce the necessity of early and longitudinal monitoring, as molecular dysregulations related to neurological functions are discernible already at the pluripotent stage, long before the clinical manifestation of severe psychopathology [52]. Ultimately, bridging clinical pharmacoresistance with transcriptomic evidence of dysregulated neuronal morphogenesis enhances the transdiagnostic understanding of sex chromosome aneuploidies [52].

While these mechanisms remain speculative, they offer plausible explanations for the limited and unpredictable efficacy of psychotropic medications in this population.

Pharmacoresistance also had profound functional implications in our patients, necessitating intensive multidisciplinary care. All three subjects required long-term follow-up by child neuropsychiatric services, multiple hospitalizations, and individualized educational and rehabilitative programs. Nevertheless, episodes of hetero-aggression, impulsive sexual behaviors, and paranoid ideation persisted, significantly impacting autonomy, adaptive functioning, and quality of life. The burden of care extended to families and healthcare systems, underscoring the need for early identification of high-risk profiles and alternative intervention strategies.

Environmental and familial dynamics are likely to modulate the expression and severity of neuropsychiatric manifestations. In Case 1, early parental separation, inconsistent paternal involvement, and sustained maternal psychological distress may have acted as psychosocial stressors, potentiating the underlying neurogenetic vulnerability. In Case 3, persistent relational and educational conflicts within the parental dyad appear to have further contributed to symptom exacerbation.

Although these influences are not systematically studied in large 47,XYY cohorts, evidence supports the role of early psychosocial adversity in modifying phenotypic expression [19,36], consistent with broader gene-environment interaction models in neurodevelopmental disorders.

Genetic comorbidities appeared to play a limited role in these cases. One patient presented with a maternally inherited 17p11.2 duplication of uncertain significance, while the others had no additional CNVs. This relative genomic simplicity suggests that the observed psychiatric burden is directly attributable to the 47,XYY karyotype rather than additional pathogenic variants.

The remarkable similarity among these patients—despite minor differences in familial background, cognitive levels, and pharmacological strategies—raises the possibility of a recognizable XYY behavioral-neuropsychiatric endophenotype. All three followed a trajectory of early developmental delay, progressive emotional and behavioral instability during adolescence, limited pharmacologic responsiveness, and increasing dependence on educational and social support. This pattern aligns with the “core plus comorbidity” model, in which a central phenotype of language and executive dysfunction is variably accompanied by psychiatric disorders depending on environmental, cognitive, and biological factors [17].

Integrating the clinical profile of 47,XYY syndrome with established data on Klinefelter syndrome (KS) facilitates a comprehensive transdiagnostic sex chromosome aneuploidy (SCA) framework. This approach supports the notion that neurodevelopmental vulnerability and psychiatric complexity represent shared mechanisms across SCAs rather than 47,XYY-specific phenomena. Both syndromes share prominent physiological markers, specifically tall stature resulting from pseudoautosomal gene overdosage, which is a consistent trait in both 47,XXY and 47,XYY populations [53]. Neurodevelopmentally, both conditions exhibit marked vulnerabilities in language acquisition and executive functions, with language delays often serving as the primary diagnostic clue [54]. While 47,XYY typically presents with a greater penetrance of social and attentional deficits, both syndromes share significantly elevated risks for ADHD and Autism Spectrum Disorder [54]. Cognitive profiles in both SCAs are frequently characterized by verbal IQ being more significantly affected than performance IQ, alongside specific deficits in working memory and inhibitory control [54].

Psychiatric complexity typically intensifies during the second decade of life, where a heightened susceptibility to mood and schizophrenia spectrum disorders becomes apparent in both groups [53,54]. The convergence of these clinical trajectories suggests that the underlying genetic architecture of SCAs predisposes individuals to similar neuropsychiatric outcomes across development. Social-emotional challenges in 47,XYY mirror the social cognition deficits well-documented in KS literature, particularly regarding the perception of affective cues [53]. These shared deficits include difficulties in interpreting facial expressions and identifying emotions, contributing to significant challenges in social integration [54]. Furthermore, both syndromes involve increased metabolic risks, such as central adiposity and type 2 diabetes, requiring routine clinical surveillance [53].

Examining Jacobs syndrome in the context of KS clinical characteristics supports a unified management approach that addresses hormonal, cognitive, and psychiatric domains [53]. However, the emerging evidence of multi-class pharmacoresistance in 47,XYY adolescents highlights a potential divergence in treatment response that warrants further study. Framing 47,XYY within the KS context underscores that long-term morbidity in both syndromes is primarily driven by neuropsychiatric complexity rather than overt somatic pathology [54]. Adolescence represents a critical window during which early developmental vulnerabilities may transition into severe psychiatric symptoms, necessitating long-term monitoring [54]. This transdiagnostic framework is essential for identifying early predictors of high-risk trajectories and optimizing rehabilitative strategies. Ultimately, the shared patterns of executive dysfunction and language impairment support a cohesive approach to neurodevelopmental interventions [54]. Such a perspective enhances our understanding of the shared genetic mechanisms governing neurodevelopmental trajectories in sex chromosome aneuploidies. Finally, this comparative approach is crucial for developing targeted interventions to mitigate the shared risks of psychiatric hospitalization and functional impairment [54].

Finally, the timing of diagnosis is noteworthy. All three patients received early genetic diagnosis (before age 10), yet this did not prevent the emergence of significant psychiatric morbidity, consistent with data suggesting that early diagnosis facilitates anticipatory guidance and access to services but does not necessarily mitigate psychiatric outcomes [49]. Early diagnosis may nonetheless allow timely educational accommodations, genetic counseling, and proactive multidisciplinary management.

Overall, these cases underscore the chronicity and complexity of care required for XYY individuals, emphasizing the need for structured clinical pathways and longitudinal management strategies. Integrated care models that combine medical, developmental, educational, and psychiatric interventions remain essential to optimize long-term outcomes [21,36].

## 4. Limitations

This narrative review provides a non-systematic account of the literature on 47,XYY syndrome, focusing particularly on psychiatric comorbidities and their management. The accompanying case series includes only three adolescents, which restricts the generalisability of clinical observations and prevents conclusions being drawn about prevalence, causality or treatment efficacy. Retrospective data collection may also be affected by incomplete documentation and variability in assessments. Furthermore, all patients were seen in a tertiary-care setting, which introduces referral bias towards more complex cases. Furthermore, the absence of standardised outcome measures restricts the systematic evaluation of treatment responses and comparisons with other cohorts. Together, these factors highlight the exploratory, descriptive nature of this work and the need for larger prospective studies with standardised longitudinal assessments.

## 5. Conclusions

This narrative review, together with a case series of adolescents with 47,XYY syndrome, highlights the marked heterogeneity and clinical complexity of the neuropsychiatric phenotype associated with this chromosomal condition. While somatic features are often mild and nonspecific, the literature consistently points to early neurodevelopmental vulnerabilities, particularly involving language, executive functioning, and attention, with high rates of ADHD, autism spectrum traits, and behavioral dysregulation.

The presented cases illustrate a shared developmental trajectory characterized by early developmental delays and a progressive worsening of emotional and behavioral symptoms during adolescence, accompanied by substantial psychiatric comorbidity and limited response to multiple psychotropic medications. This pattern suggests that pharmacoresistance may represent an underrecognized but clinically relevant feature in a subset of individuals with 47,XYY syndrome.

Overall, the combined evidence supports the existence of a potential 47,XYY-specific neuropsychiatric phenotype in 47,XYY syndrome, in which core neurodevelopmental impairments may interact with developmental and environmental factors to shape complex psychiatric outcomes. Although early diagnosis facilitates access to support and interventions, it does not fully prevent the emergence of severe psychopathology, underscoring the need for long-term monitoring.

These findings emphasize the importance of integrated, multidisciplinary management strategies that extend beyond pharmacological treatment and incorporate rehabilitative, educational, and family-centered interventions. Future studies should focus on identifying early markers of high-risk trajectories and on developing more targeted therapeutic approaches for this population.

## Figures and Tables

**Table 1:** Case 1 Pharmacological history.

Age	Pharmacological Treatment	Clinical Outcome/Notes
10y	Aripiprazole (monotherapy), later combined with valproic acid, chlorpromazine, and lithium	Discontinued at age 15 due to inefficacy
12y9m	Risperidone	Partial benefit; weight gain/hyperphagia. Discontinued at age 14y1m, reintroduced at age 14y7m and discontinued at age 15y8m
14y1m	Valproic acid + Chlorpromazine + Aripiprazole	Limited benefit
14y5m	Valproic acid + Aripiprazole + Lithium	Ineffective, worsening behavior
14y6m	Methylphenidate	Paradoxical excitability, discontinued at age 14y10m
14y8m	Clozapine	Discontinued due to side effects at age 14y9m (hyperthermia, hypotension, tachycardia)
15y2m	Valproic acid + Lithium + Olanzapine + Chlorpromazine	Partial benefit
15y11m	Add-on Haloperidol + Lorazepam+ Biperidene (tremors) to lithium, and olanzapine.	
16y6m	Clozapine re-challenge; olanzapine and lorazepam gradually discontinued.	Partial benefit
15y8m	Allopurinol	Additional therapy for hyperuricemia and lower limb myalgia

**Table 2:** Case 2 Pharmacological history.

Age	Pharmacological Treatment	Clinical Outcome/Notes
7y9m	Methylphenidate	Initiated for ADHD. Discontinued at age 13 due to lack of benefit
9y	Risperidone + Methylphenidate	Partial benefit; Risperidone discontinued at age 11y8m due to significant weight gain
11y8m	Aripiprazole + Lithium + Methylphenidate	Mild improvement in irritability;
13y4m	Chlorpromazine + Aripiprazole + Lithium	Chlorpromazine added for worsening dysregulation, providing partial benefit
14y8m	Lurasidone + Chlorpromazine + Lithium	Lurasidone substituted for aripiprazole to address weight concerns, thought content changes, and depressed mood. Limited clinical effect led to gradual dose reduction, while chlorpromazine was titrated up to 300 mg/day.

**Table 3:** Case 3 Pharmacological history.

Age	Pharmacological Treatment	Clinical Outcome/Notes
6y	Sodium Valproate	Limited efficacy
6y4m	Risperidone + Sodium Valproate	Initially effective; hyperprolactinemia and weight gain
7y	Aripiprazole + Sodium Valproate	Ineffective, progressive worsened behavior
8y10m	Lithium carbonate + Aripiprazole	Partial stabilization
9y6m	Sodium Valproate + Olanzapine + Lithium carbonate	Partial reduction in anger outbursts
9y8m	Risperidone + Sodium Valproate + Lithium carbonate (during hospitalization)	Partial benefit
9y10m	Chlorpromazine + Sodium Valproate	Persistent moderate stabilization, and subsequently photosensitivity and sialorrhea
13y10m	Risperidone + Sodium Valproate	

## Data Availability

The original contributions presented in this study are included in the article. No additional datasets were generated for this study. Further inquiries can be directed to the corresponding author due to ethical and privacy restrictions.
